# The Invention of Spectacles

**Published:** 1872-11

**Authors:** 


The Invention of Spectacles.—On a tombstone in Florence is this inscription: "Here lies Salvino Armato d'Armati, of Florence, the inventor of spectacles. May God pardon his sins. The year 1318.—*Ex.*"

